# Chaperonin 60 sustains osteoblast autophagy and counteracts glucocorticoid aggravation of osteoporosis by chaperoning RPTOR

**DOI:** 10.1038/s41419-018-0970-6

**Published:** 2018-09-17

**Authors:** Wei-Shiung Lian, Jih-Yang Ko, Yu-Shan Chen, Huei-Ching Ke, Shin-Long Wu, Chung-Wen Kuo, Feng-Sheng Wang

**Affiliations:** 1 Core Laboratory for Phenomics and Diagnostic, Kaohsiung Chang Gung Memorial Hospital, Kaohsiung, Taiwan; 2 Department of Medical Research, Kaohsiung Chang Gung Memorial Hospital, Kaohsiung, Taiwan; 3 Department of Orthopedic Surgery, Kaohsiung Chang Gung Memorial Hospital, Kaohsiung, Taiwan; 4 Graduate Institute of Clinical Medical Science, Chang Gung University College of Medicine, Kaohsiung, Taiwan

## Abstract

Glucocorticoid excess medication interrupts osteoblast homeostasis and exacerbates bone mass and microstructure loss ramping up the pathogenesis of osteoporotic disorders. Heat shock protein 60 (HSP60) is found to maintain protein function within cellular microenvironment upon encountering detrimental stress. In this study, we revealed that supraphysiological dexamethasone decreased HSP60 expression along with deregulated autophagy in osteoblasts cultures. This chaperonin is required to sustain autophagic markers Atg4, and Atg12 expression, LC3-II conversion, and autophagic puncta formation, and alleviated the glucocorticoid-induced loss of osteogenic gene expression and mineralized matrix accumulation. Regulator-associated protein of mTOR complex 1 (RPTOR) existed in HSP60 immunoprecipitate contributing to the HSP60-promoted autophagy and osteogenesis because knocking down RPTOR impaired autophagic influx and osteogenic activity. HSP60 shielded from RPTOR dysfunction by reducing the glucocorticoid-induced RPTOR de-phosphorylation, aggregation, and ubiquitination. In vivo, forced RPTOR expression attenuated the methylprednisolone-induced loss of osteoblast autophagy, bone mass, and trabecular microstructure in mice. HSP60 transgenic mice displayed increased cortical bone, mineral acquisition, and osteoblast proliferation along with higher osteogenesis of bone marrow mesenchymal cells than those of wild-type mice. HSP60 overexpression retained RPTOR signaling, sustained osteoblast autophagy, and compromised the severity of glucocorticoid-induced bone loss and sparse trabecular histopathology. Taken together, HSP60 is essential to maintain osteoblast autophagy, which facilitates mineralized matrix production. It fends off glucocorticoid-induced osteoblast apoptosis and bone loss by stabilizing RPTOR action to autophagy. This study offers a new insight into the mechanistic by which chaperonin protects against the glucocorticoid-induced osteoblast dysfunction and bone loss.

## Introduction

Glucocorticoid medication is widely prescribed to lessen immune irregularity and malignant hematological disorders, like allergy, arthritis, and leukemia^1^. Chronic glucocorticoid use damages survival and anabolism of bone cells aggravating excessive bone loss and microarchitecture porosity, prominent deleterious effects notorious for osteoporosis development^2,3^. Organelle deregulation and protein dysfunction speed up osteoblast apoptosis contributing to the progression of glucocorticoid-mediated osteoporosis. For example, high concentrations of glucocorticoid disrupt endoplasmic reticulum^4^ and deteriorates mitochondrial integrity^5^ inducing a burst of reactive oxygen radicals that drastically impedes osteoblast viability. Increased E3 ubiquitin ligase signaling ramps up glucocorticoid-induced osteopenia in mice^6^. The mechanism underlying the glucocorticoid-impaired intracellular homeostasis and organelle machinery in osteoblasts upon glucocorticoid stress has not been well investigated.

Autophagy is a sophisticated intracellular program, including progressive autophagosome formation and lysosomal degradation, responsible for removing abandoned organelle or proteins to maintain regular physiological activity of cells upon experiencing adverse conditions^7^. As regards the biological role of autophagy in skeletal metabolism, knockout mice deficient in autophagic vesicle builder Atg5 in osteoblasts show poor mineralization capacity and low bone mineral density^8^. Mice lacking Atg5 and Atg7 in chondrocytes exhibit bone undergrowth^9^. Poor autophagy slows down bone development in fibroblast growth factor knockout mice^10^. In addition, mice lacking upstream autophagy regulator mammalian target of rapamycin complex 1 (mTORC1) in osteoblasts display low bone mass and meager trabecular microstructure^11^, whereas osteoclast-specific mTORC1 knockout mice reveal high bone mineral density owing to impaired osteoclastic resorption capacity^12^. The role. of mTORC1 signaling components in the progression of glucocorticoid-induced osteoporosis remains elusive.

Heat shock protein 60 (HSP60) is a mitochondrial chaperonin, stabilizing protein function through chaperoning unfolded proteins into a folded state^13^. HSP60 knockout causes intestinal epithelial stem cells to lose stemness and proliferation capacity^14^. Decreased HSP60 impairs mitochondrial integrity worsening the fused sarcoma-associated neurodegenerative disorders^15^, whereas increasing this chaperonin promotes mitochondrial energy expenditure of murine skeletal muscle during endurance training^16^. Upregulating HSP60 facilitates alveolar macrophage autophagy to augment inflammatory responses in lung tissue upon ischemia and reperfusion^17^. We previously found that mice overexpressing HSP60 exhibited minor responses to chondrocyte apoptosis and knee osteoarthritis progression^18^. Silencing HSP60 expression induced bone loss in rats^19^. Given that HSP60 participated in bone metabolism, we hypothesized that it may regulate osteoblast autophagy during the pathogenesis of glucocorticoid excess-induced osteoporotic skeletons.

This study is undertaken to investigate molecular events underlying HSP60 action to glucocorticoid-impaired osteoblast autophagy and osteogenic differentiation, and utilized HSP60 transgenic mice to verify its regulatory functions to osteoblast autophagy, survival, and bone mass homeostasis in long-term glucocorticoid excess-treated skeletal tissue.

## Results

### HSP60 loss worsened glucocorticoid-inhibited autophagy and osteogenesis

**Fig. 1:**
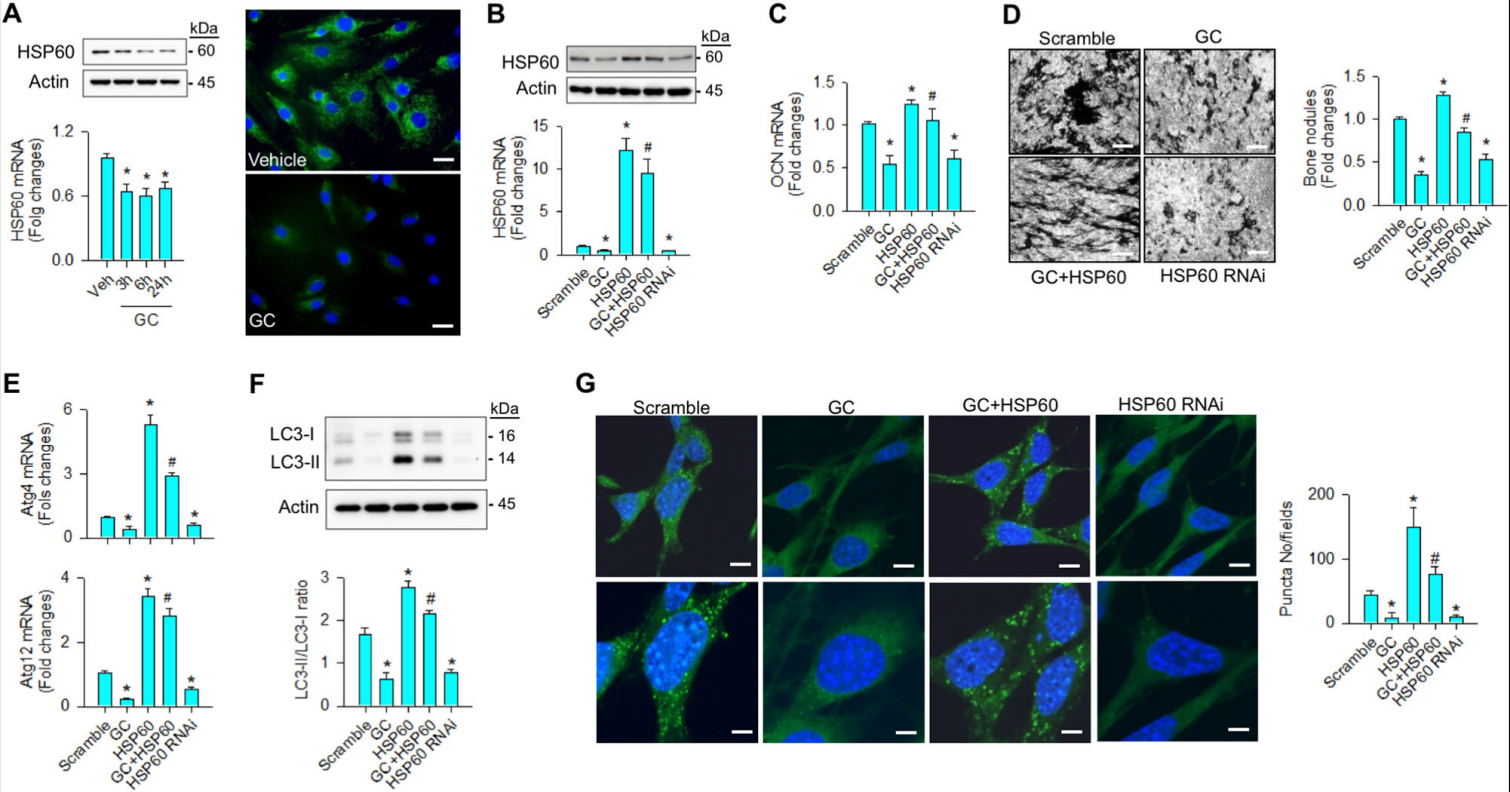
Analyses of HSP60-mediated autophagy and differentiation of osteoblasts. **a** Glucocorticoid reduced mRNA expression, protein levels, and immunofluorescence of HSP60 in osteoblasts. Scale bar, 8 μm. **b** Forced HSP60 expression attenuated the glucocorticoid-induced loss of (**c**) osteocalcin expression and **d** mineralized matrix accumulation. Scale bar, 40 μm. Increasing HSP60 attenuated the glucocorticoid-mediated loss of (**e**) Atg4, and Atg12 expressions, (**f**) LC3-II levels, LC3-II/LC3-I ratio, and **g** autophagic puncta formation. Scale bars, 6 μm (upper panels) and 12 μm (lower panels). Knocking down HSP60 decreased autophagic marker expressions, autophagic vesicle formation, and mineralized matrix production of osteoblasts. Experiments results are expressed as mean ± standard error. Asterisks (*) resemble a significant difference (*P* < 0.05) vs. vehicle or scramble group, and hashtag (#) indicates a distinguishable difference (*P* < 0.05) vs. glucocorticoid-treated group. Veh, vehicle; GC, glucocorticoid

To examine whether HSP60 affected autophagy in glucocorticoid-stressed osteoblasts, cell cultures were incubated in osteogenic medium containing 1 μM dexamethasone, an in vitro model that mimics glucocorticoid excess-induced bone loss^20^. Dexamethasone significantly reduced HSP60 mRNA expression and protein levels along with slight HSP60 immunofluorescence in cytoplasmic compartment as compared with vehicle group (Fig. 1a). Forced HSP60 expression (Fig. 1b) lessened the glucocorticoid-induced loss of osteogenic activities, like osteocalcin expression (Fig. 1c) and von Kossa staining-positive mineralized matrix formation (Fig. 1d), whereas knocking down HSP60 significantly reduced baseline osteogenesis as compared with scrambled controls (Fig. 1b, c, d).

With regard to autophagic activity, glucocorticoid or HSP60 interference significantly decreased autophagy markers Atg4, and Atg12 expressions (Fig. 1e), concomitant with reduced LC3-II levels, LC3-II/LC3-I ratio (Fig. 1f), and autophagic puncta as evident from fluorescent probe monodansylcadaverin for autophagic vacuoles (Fig. 1g). Increasing HSP60 attenuated the glucocorticoid-mediated loss of autophagic marker expressions, LC3-II abundances, LC3-II/LC3-I ratio, and autophagic vesicles (Fig. 1e, f, g), which was suggestive of HSP60 maintaining autophagic influx in osteoblasts upon glucocorticoid stress.

### HSP60 regulated RPTOR signaling

**Fig. 2:**
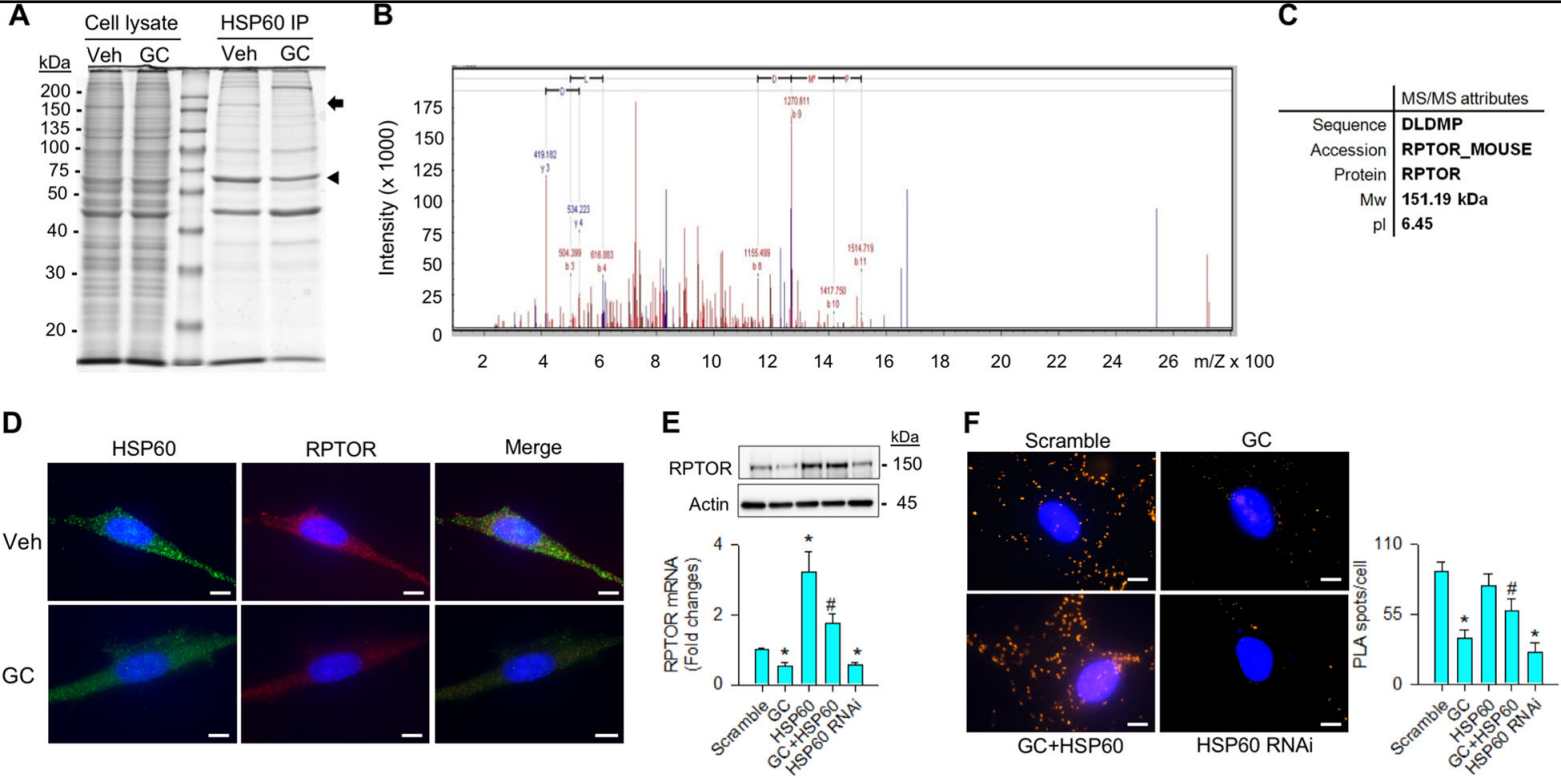
Analyses of HSP60 modulation of RPTOR signaling in osteoblasts. **a** A protein corresponding to 150 kDa (arrow) along with HSP60 (arrowhead) existed in HSP60 immunoprecipitates. Images of cell lysate indicate equal load of specimens for electrophoresis. **b** Tandem mass spectrum and **c** attributes of peptide homologous to RPTOR. **d** Weak HSP60 (green) and RPTOR (red) immunofluorescence in glucocorticoid-stressed osteoblasts. Scale bars, 16 μm. **e** Increasing HSP60 attenuated the glucocorticoid-induced loss of RPTOR mRNA expression and protein levels. **f** Images of HSP60 and RPTOR-ligated complexes. Forced HSP60 expression increased HSP60 and RPTOR ligation reaction in osteoblasts, whereas HSP60 knockdown reduced the reaction. Scale bar, 16 μm. Experiments results are expressed as mean ± standard error. Asterisks (*) resemble a significant difference (*P* < 0.05) vs. scramble group, and hashtag (#) indicates a distinguishable difference (*P* < 0.05) vs. glucocorticoid-treated group

To understand how HSP60 retained autophagy in glucocorticoid-stressed osteoblasts, HSP60 immunoprecipitates were isolated for electrophoresis. Sodium dodecyl sulfate–polyacrylamide gel electrophoresis analysis revealed that a protein corresponding to ~ 150 kDa was decreased in the glucocorticoid-treated cells (Fig. 2a). The protein band of interest was cut for in-gel trypsin digestion and liquid chromatography to characterize peptide sequence. The protein was highly homologous to regulatory-associated protein of mTOR (RPTOR) as evident from tandem mass spectrum (Fig. 2b, c). Consistently, glucocorticoid-treated osteoblasts showed weak HSP60 (green) and RPTOR (red) immunofluorescence (Fig. 2d). In addition, glucocorticoid or HSP60 RNAi treatment significantly depleted RPTOR expressions. Forced HSP60 expression lessened the extent of glucocorticoid-induced RPTOR loss (Fig. 2e). Proximity ligation analyses also confirmed an interaction between HSP60 and RPTOR as evident from abundant protein ligated complexes displaying fluorescent spots in the scramble control-transfected and HSP60-transfected cells, whereas glucocorticoid stress or HSP60 interference inhibited the reaction (Fig. 2f).

### RPTOR attenuated the glucocorticoid suppression of autophagy and osteogenesis

**Fig. 3:**
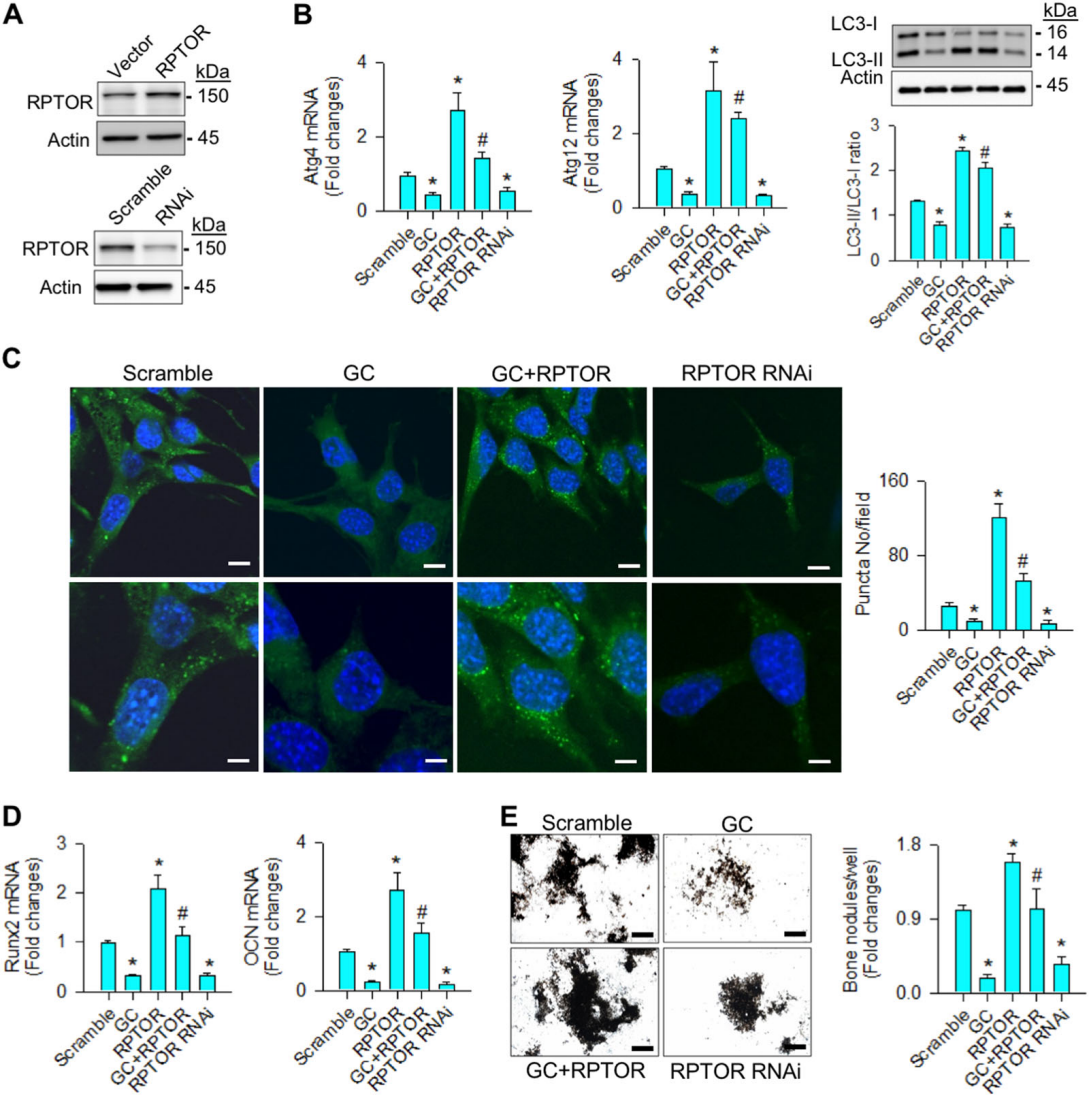
RPTOR promoted autophagy and osteogenic activities. **a** RPTOR levels in RPTOR cDNA- and RNAi-transfected osteoblasts. **b** Increasing RPTOR alleviated the glucocorticoid-induced loss of Atg4, and Atg12 expressions, LC3-II levels, LC3-II/LC3-I ratio, and **c** autophagic puncta. Scale bars, 8 μm (upper panels) and 16 μm (lower panels). It also restored **d** Runx2 and osteocalcin expression, and **e** mineralized nodule formation. Knocking down RPTOR impaired autophagic marker expression, autophagic vesicle formation, and osteogenic activity. Scale bar, 46 μm. Experiments results are expressed as mean ± standard error. Asterisks (*) resemble a significant difference (*P* < 0.05) vs. scramble group, and hashtag (#) indicates a distinguishable difference (*P* < 0.05) vs. glucocorticoid-treated group

To test whether RPTOR signaling changed glucocorticoid-inhibited autophagy or osteogenesis, cell cultures were transfected with RPTOR complementary DNA (cDNA) or RNA interference (RNAi). Gain of RPTOR function (Fig. 3a) attenuated glucocorticoid-inhibited Atg4, and Atg12 expressions, LC3-II concentrations, LC3-II/LC3-I ratio (Fig. 3b), and autophagic puncta formation (Fig. 3c). Interfering with RPTOR significantly decreased baseline expressions of autophagic marker along with very few autophagosome vesicles (Fig. 3a, b, c). In addition, forced RPTOR expression alleviated the glucocorticoid-induced loss of osteogenic markers Runx2 and osteocalcin expressions (Fig. 3d), and mineralized nodules accumulation (Fig. 3e). Knocking down RPTOR also significantly downregulated baseline osteogenic activities (Fig. 3d, e).

### HSP60 ameliorated glucocorticoid-induced loss of RPTOR stability

**Fig. 4:**
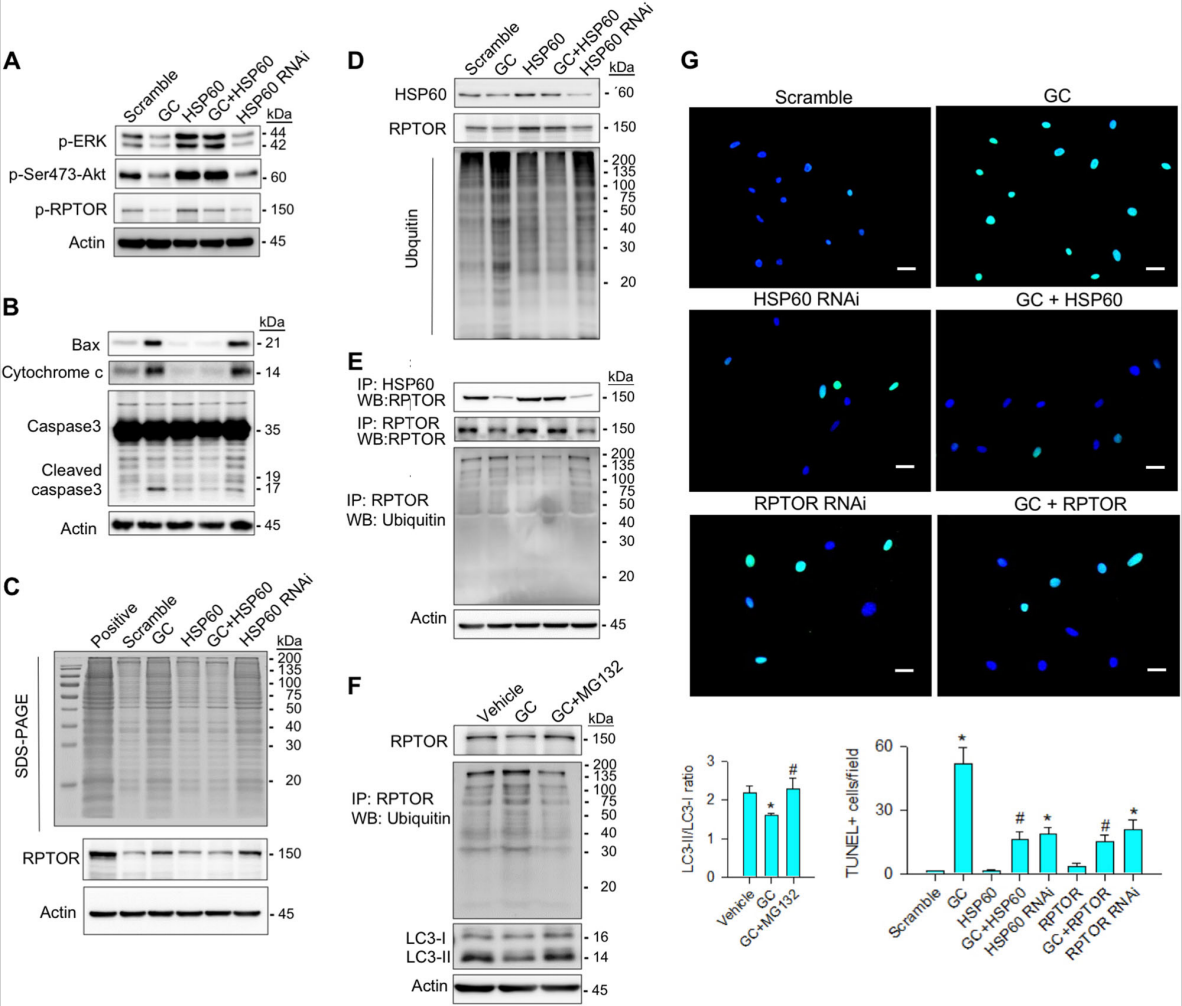
HSP60 protected RPTOR from the glucocorticoide-phosphorylation, aggregation, and ubiquitination. **a** HSP60 restored the glucocorticoid-mediated loss of phosphorylated ERK, phosphorylated Ser473-Akt, and phosphorylated RPTOR abundances. **b** It attenuated glucocorticoid-enhanced Bax, cytochrome c, and cleaved caspase3 levels. **c** Forced HSP60 expression alleviated the glucocorticoid elevation of protein aggregation along with decreased RPTOR aggregation. Actin immunoblots indicate equal amount of cell lysates pipetted for protein aggregation assay. **d**, **e** HSP60 overexpression reduced the glucocorticoid-upregulated levels of ubiquitinated proteins and RPTOR. Knocking down HSP60 impaired RPTOR phosphorylation but increased RPTOR aggregation and ubiquitination. **f** MG132 attenuated the glucocorticoid-induced RPTOR ubiquitination and restored RPTOR and LC3-II levels and LC3-II/LC3-I ratio. **g** Images of TUNEL staining in osteoblasts. HSP60 and RPTOR attenuated glucocorticoid-mediated apoptosis. Scale bar, 85 μm. Experiments results are expressed as mean ± standard error. Asterisks (*) resemble a significant difference (*P* < 0.05) vs. scramble group, and hashtag (#) indicates a distinguishable difference (*P* < 0.05) vs. glucocorticoid-treated group

We examined whether HSP60 affected RPTOR stability because it is found to modulate protein function through post-translational modification^21,22^. Glucocorticoid or HSP60 RNAi decreased levels of phosphorylated ERK, phosphorylated Ser473-Akt, and phosphorylated RPTOR (Fig. 4a), whereas concentrations of Bax, cytochrome c, and cleaved caspase3 were increased (Fig. 4b). Increasing HSP60 retained abundances of these three phosphorylated proteins and mitigated the glucocorticoid-upregulated apoptotic signaling (Fig. 4a, b).

Given that unstable proteins are inclined to form aggregates^21^, we examined whether glucocorticoid or HSP60 affected protein aggregation. To prepare positive controls, acetone was added to cell lysate to induce protein aggregation. Aggregated proteins in cell lysates were ultra-centrifuged and electrophoresed. Glucocorticoid or HSP60 knockdown increased levels of aggregated proteins. Increased RPTOR also existed in the aggregated proteins (Fig. 4c). With ubiquitin antibody probing, glucocorticoid, or HSP60 RNAi upregulated concentrations of ubiquitinated proteins (Fig. 4d) and ubiquitinated RPTOR (Fig. 4e). Increasing HSP60 expression attenuated glucocorticoid-enhanced levels of aggregated and ubiquitinated proteins along with reduced abundances of aggregated and ubiquitinated RPTOR (Fig. 4c, d, e). We tested if inhibiting RPTOR ubiquitination affected autophagy. Inactivating ubiquitin by proteasome inhibitor MG132 attenuated levels of ubiquitinated RPTOR and lessened the glucocorticoid-induced loss of RPTOR, LC3-II levels, and LC3-II/LC3-I ratio (Fig. 4f). Furthermore, glucocorticoid or HSP60 RNAi or RPTOR RNAi significantly increased osteoblasts apoptosis compared with scrambled controls as evident from fluorescent TUNEL staining, whereas overexpressing HSP60 or RPTOR attenuated glucocorticoid-induced apoptosis (Fig. 4g).

### RPTOR alleviated glucocorticoid-induced bone deterioration

**Fig. 5:**
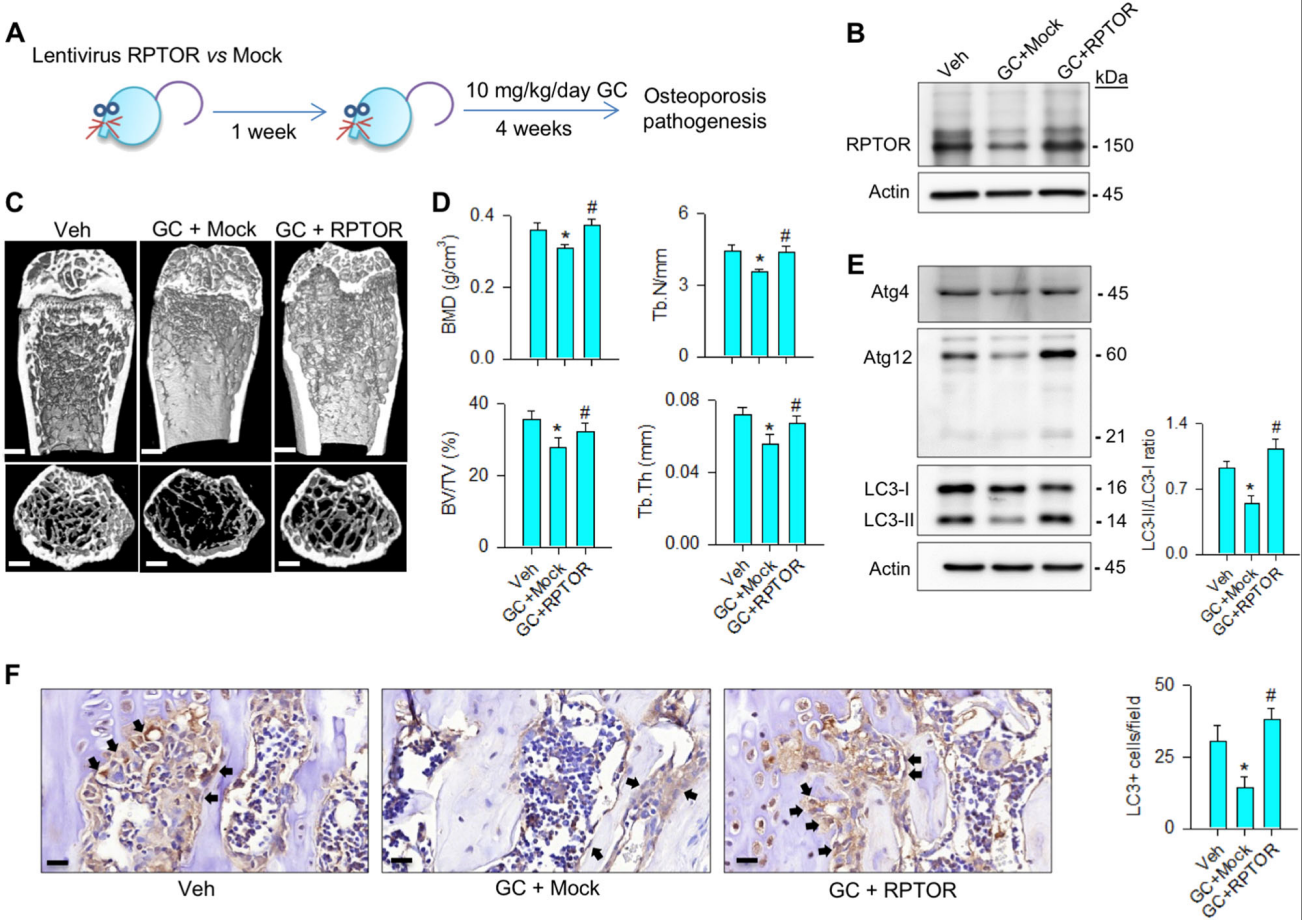
Forced RPTOR expression attenuated the glucocorticoid-induced bone loss. **a** Schematic drawing for lentivirus RPTOR treatment for glucocorticoid-treated mice. **b** RPTOR treatment reduced the methylprednisolone-induced loss of RPTOR levels. **c** μCT images of proximal tibiae showed sparse trabecular bone structure upon glucocorticoid treatment, whereas abundant bone microstructure remained in the RPTOR-treated group. Scale bar, 5 mm. **d** Increasing RPTOR expression alleviated the methylprednisolone-induced loss of BMD, BV/TV, Tb.N, and Tb.Th. **e** This treatment also retained Atg4, Atg12, LC3-II levels, and LC3-II/LC3-I ratio. **f** LC3 immunostaining (arrows) in osteoblasts along trabecular bone was decreased upon glucocorticoid treatment, whereas forced RPTOR expression improved LC3 immunoreactivity. Scale bar, 10 μm. Experiments were performed on six mice, and results are expressed as mean ± standard error. Asterisks (*) resemble a significant difference (*P* < 0.05) vs. vehicle-treated group, and hashtag (#) indicates a distinguishable difference (*P* < 0.05) between the methylprednisolone-treated group vs. RPTOR group. Veh, vehicle; GC, glucocorticoid

Given that RPTOR signaling promoted osteoblast function in vitro, we verified whether it affected glucocorticoid-induced bone loss. Mice were injected with lentivirus RPTOR and followed by methylprednisolone treatment for 4 consecutive weeks (Fig. 5a). It attenuated the glucocorticoid-induced loss of RPTOR levels in bone tissue (Fig. 5b). Glucocorticoid-induced sparse trabecular microstructure (Fig. 5c) and significantly decreased bone mineral density (BMD), trabecular bone volume (BV/TV), trabecular number (Tb.N), and trabecular thickness (Tb.Th) (Fig. 5d). Increasing RPTOR lessened the severity of glucocorticoid-mediated loss of bone mass, microarchitecture, and morphometric characteristics (Fig. 5c, d). It also retained autophagy markers Atg4, Atg12, LC3-II levels, and LC3-II/LC3-I ratio (Fig. 5e), and preserved LC3 immunostaining in osteoblasts along trabecular bone in glucocorticoid-treated skeletons (Fig. 5f).

### HSP60 overexpression attenuated the glucocorticoid-induced bone loss

**Fig. 6:**
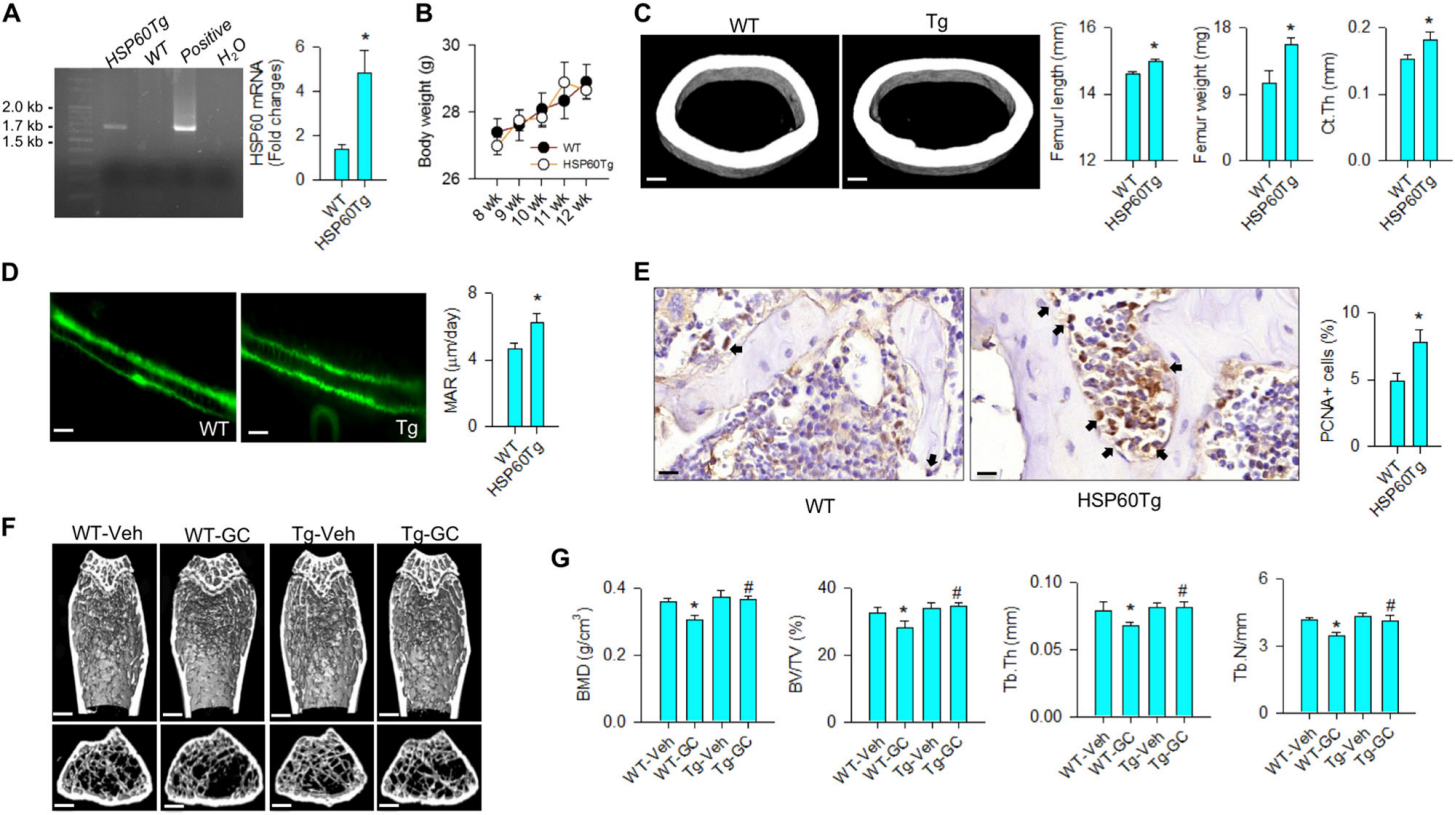
Analyses of bone mass and microstructure of HSP60Tg mice. **a** HSP60Tg mice carried the construct and showed increased HSP60 expression of bone tissue. Images of agarose electrophoresis of positive and H_2_O indicate positive control (plasmid construct) corresponding to 1.7 kbp and negative control, respectively. **b** Body weight of HSP60Tg was comparable to wild-type mice. **c** HSP60Tg mice showed significant increases in femur weight, femur length, cortical bone thickness (scale bar, 50 μm), and **d** calcein-labeled bone mineral acquisition (scale bar, 30 μm). **e** Osteoblasts in bone tissue in HSP60Tg mice displayed strong PCNA immunostaining (arrows). Scale bar, 10 μm. **f** μCT images of tibiae. Glucocorticoid-treated wild-type mice showed sparse trabecular microarchitecture, whereas abundant trabecular bone remained in glucocorticoid-treated HSP60Tg mice. Scale bar, 5 mm. **g** HSP60Tg mice displayed minor responses to the glucocorticoid-induced loss of BMD, BV/TV, Tb.Th, and Tb.N. Experiments were performed on six mice, and results are expressed as mean ± standard error. Asterisks (*) resemble a significant difference (*P* < 0.05) vs. wild-type group, and hashtag (#) indicates a distinguishable difference (*P* < 0.05) between the group of interest vs. glucocorticoid-treated wild-type group

To understand whether increasing HSP60 changed glucocorticoid-induced bone loss, mice that overexpressed HSP60 (HSP60Tg) driven by PGK promoter were bred for study^18^. HSP60Tg mice carried the 1.7 kbp construct of interest; HSP60 expression was significantly increased in bone tissue (Fig. 6a). They gained body weight comparable to wild-type mice throughout the study (Fig. 6b). With respect to skeletal phenotypes, HSP60 overexpression significantly increased bone length and bone weight along with thicker cortical bone as compared with wild-type mice (Fig. 6c). Bone mineral acquisition and osteoblast growth were also promoted in HSP60Tg mice as evident from fluorescent calcein labeling (Fig. 6d) and proliferating cell nuclear antigen immunostaining (Fig. 6e), respectively. Of interest, abundant trabecular bone microstructure remained in HSP60Tg mice at 4 weeks after methylprednisolone stress, whereas decreased trabecular architecture existed in methylprednisolone-treated wild-type mice (Fig. 6f). Consistently, the severity of glucocorticoid-induced loss of morphometric characteristics BMD, BV/TV, Tb.Th, and Tb.N was significantly compromised in HSP60Tg mice (Fig. 6g).

### HSP60 reduced glucocorticoid-mediated loss of autophagy and osteogenesis

**Fig. 7:**
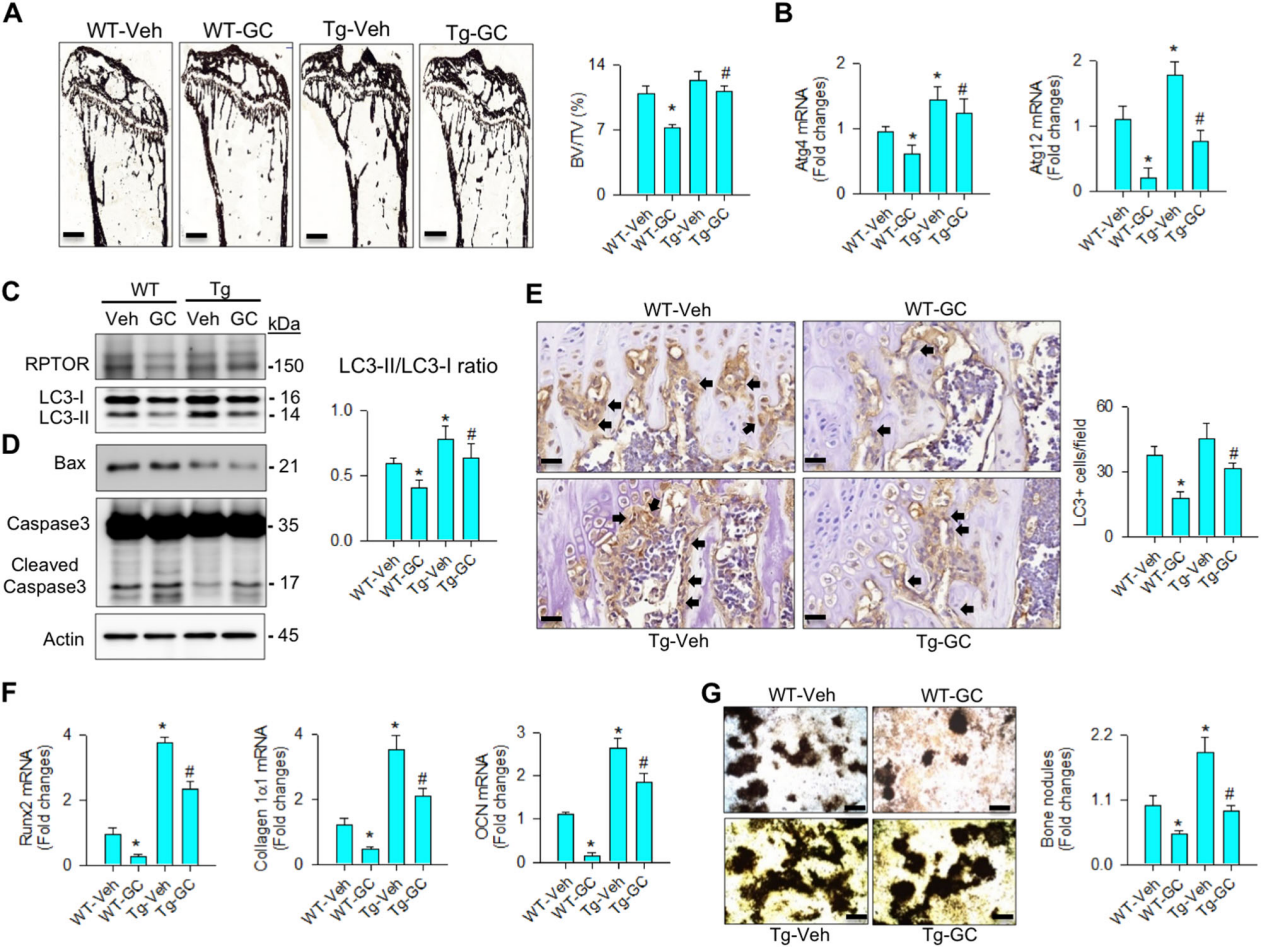
Analyses of bone histomorphomertry and osteogenic activity of bone marrow mesenchymal cells. HSP60Tg mice showed minor responses to the glucocorticoid-induced loss of (**a**) BV/TV (scale bar, 120 μm), **b** Atg4, and Atg12 expressions, (**c**) RPTOR, and LC3-II levels, and LC3-II/LC3-I ratio. HSP60 overexpression (**d**) reduced levels of Bax and cleaved caspase3 but (**e**) increased LC3 immunostaining in osteoblasts (arrows) in glucocorticoid-treated bone tissue. Scale bar, 8 μm. HSP60 overexpression mitigated the glucocorticoid-induced loss of **f** Runx2, collagen 1α1, and osteocalcin expressions, and **g** mineralized nodule formation of bone marrow mesenchymal cells. Scale bar, 46 μm. Experiments were performed on six mice, and results are expressed as mean ± standard error. Asterisks (*) resemble a significant difference (*P* < 0.05) vs. wild-type group, and hashtag (#) indicates a distinguishable difference (*P* < 0.05) between the group of interest vs. glucocorticoid-treated wild-type group

We verified whether HSP60 overexpression changed bone histology or osteoblast autophagy in skeletal tissue at 4 weeks after methylprednisolone treatment. Glucocorticoid escalated trabecular bone loss histopathology and significantly decreased BV/TV in wild-type mice. These adverse effects were significantly attenuated in HSP60Tg mice (Fig. 7a). Furthermore, HSP60 overexpression retained Atg4, and Atg12 expression (Fig. 7b), and LC3-II concentration along with increased LC3-II/LC3-I ratio (Fig. 7c) but decreased Bax and cleaved caspase3 levels (Fig. 7d). It also alleviated the glucocorticoid-induced loss of LC3 immunostaining in osteoblasts (Fig. 7e).

To verify whether HSP60 changed osteogenic differentiation capacity, primary bone marrow mesenchymal cells were isolated and incubated in osteogenic medium. In the wild-type group, glucocorticoid significantly inhibited Runx2, collagen 1α1, and osteocalcin expressions (Fig. 7f), and mineralized matrix synthesis (Fig. 7g). The extent of glucocorticoid-inhibited osteogenesis was significantly lessened in HSP60Tg mice (Fig. 7f, g).

## Discussion

Autophagy is involved in disposal of unwanted molecules to harmonize intracellular microenvironment, enabling cells to overcome deleterious conditions^7^. Deregulated autophagy ratchets up the pathogenesis of skeletal and arthritic disorders. For example, low autophagic breakdown of misfolded collagen worsens osteogenesis imperfecta in mice^23^. Overacted autophagy mediates metal implant particle-induced osteolysis^24^. Proteasome inhibitor bortezomib-suppressed autophagy weakens bone metastasis capacity of multiple myeloma cells^25^. Poor osteoblast autophagy of crystal urate increases chronic gout development^26^. Aberrant autophagy hinders osteogenesis of bone marrow mesenchymal stem cells worsening diabetes-induced bone mass loss^27^. The contribution of osteoblast autophagy to glucocorticoid-induced excessive bone catabolism has not been well defined. This study is the first uncovering HSP60 action indispensable in osteoblast autophagy for protecting skeletal tissue from glucocorticoid-induced osteoporosis. It also throws light on new mechanistic events by which mitochondrial chaperonin pathway preserves RPTOR function to autophagic influx within osteoblast microcompartment facilitating bone tissue homeostasis and microstructure integrity.

Analyses of osteoblast culture models revealed that HSP60 was required to osteogenesis because silencing this mitochondrial chaperonin largely led to mineralized matrix synthesis below baseline, overexpressing it enabled cell cultures to uphold osteogenic differentiation capacity upon supraphysiological glucocorticoid-mediated adverse stress. Increasing evidence reveals that high dose of therapeutic glucocorticoid disturbs mitochondrial machinery provoking cell damage or tissue deterioration, like skeletal muscle wasting^28^, necroptosis of acute lymphoblastic leukemic cells^29^, and fat overproduction of adipose tissue^30^. Loss of HSP60 function also perturbs extracellular matrix metabolism and amplifies apoptotic programs^31,32^. This study showed that HSP60 maintained autophagic influx beneficial for fending off glucocorticoid-induced apoptosis and mineralized matrix underproduction, indicating that glucocorticoid stress cutoff a fine-tuned organelle crosstalk between autophagosome and mitochondria aggravating osteoblast dysfunction. These interesting findings prompted us to decipher the molecular event by which HSP60 regulated osteoblast autophagy.

Of note, arrays of analyses uncovered that RPTOR interplayed with HSP60 protecting osteoblasts against the glucocorticoid-impaired autophagy and differentiation. The regulatory action of RPTOR to skeletal tissue metabolism and osteoporosis pathogenesis seems like inconclusive. For example, bone marrow mesenchymal stem cells from RPTOR knockout mice favorably turn out to be osteogenic lineages^33^. On the contrary, bone tissue-specific RPTOR knockout mice show bone underdevelopment^34^ and poor osteogenic activity of preosteoblasts^11^. Activation of mTORC1 prevents from osteoporosis progression in mice overexpressing Wnt1 in osteocytes^35^. We uncovered its role in preserving osteoblast function and bone integrity because increasing RPTOR alleviated the severity of glucocorticoid-induced bone mass loss and microstructure deterioration. Together with our findings and other groups’ investigations, this molecule appeared to serve different functions depending on bone cell types and deleterious stresses impacted on skeletal tissue. Given that HSP60 interacted with RPTOR, further experiments were performed to delineate in detail how HSP60 warded off the glucocorticoid-induced RPTOR loss.

Profound results of immunoprecipitation analysis revealed that HSP60 sustained RPTOR function by controlling multiple post-translational modification reactions of RPTOR, like phosphorylation, aggregation, and ubiquitination. HSP60 is found to convert mitochondrial proteins into a folding status, which drives them away from aggregation^36,37^. It also affects kinase phosphorylation^38^ and cellular protein degradation^39^. In this study, we conveyed a new insight into chaperone machinery protective against the glucocorticoid-mediated RPTOR dysfunction. In addition to RPTOR stability, analyses of increased survival regulators ERK and Akt, and decreased pro-apoptotic signaling Bax and cytochrome c release consolidated the experimental findings showing that HSP60 facilitated osteoblast survival. Investigations were in agreement with other groups’ studies demonstrating that Akt and ERK pathways phosphorylate RPTOR facilitating mTOR1 activity and cell growth^40,41^. Although it warrants further analysis in the future to characterize how chaperonin activates these regulators, changes in various signaling pathways indicated that multiple reactions within intracellular environment participated in stabilizing RPTOR to curtail the glucocorticoid excess-induced loss of osteogenic activity.

Intriguing findings were that HSP60Tg mice showed moderate bone overgrowth phenotypes like thickened cortical bone and increased bone mineral accretion, concomitant with intensive osteoblast proliferation. These anabolic effects kept skeletal tissue away from methylprednisolone excess-induced bone mineral density loss and trabecular porosity histopathology. Consistent with the analyses of in vitro osteoblast models, overexpressing HSP60 improved RPTOR signaling and autophagy to delay glucocorticoid-mediated osteoblast dysfunction. Collective investigations of sustained osteoblast autophagy and high osteogenic differentiation of bone marrow mesenchymal cells in HSP60Tg mice underpinned the bone anabolic function of HSP60 able to drag the glucocorticoid excess-mediated bone deterioration.

**Fig. 8:**
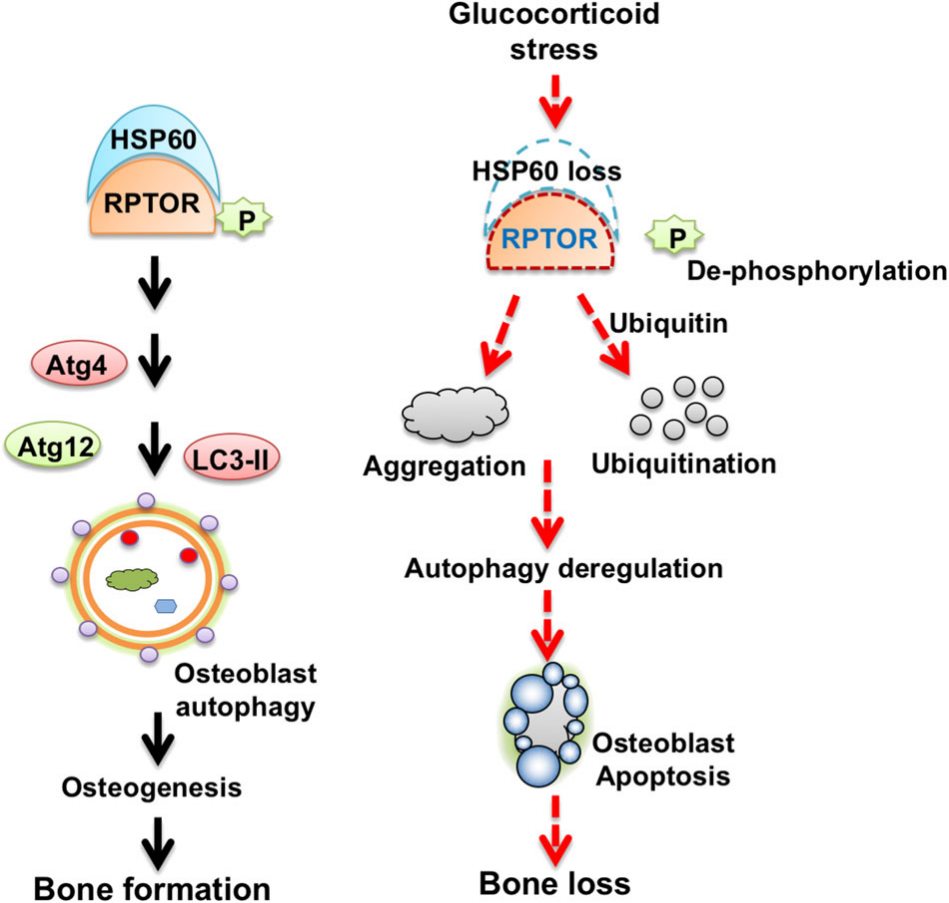
Schematic drawings for HSP60 and RPTOR protection against glucocorticoid deregulation of osteoblast autophagy and bone mass. HSP60 is required to maintain osteoblast autophagy and osteogenesis. Glucocorticoid induces HSP60 loss escalating RPTOR de-phosphorylation, aggregation, and ubiquitination to deteriorate osteoblast autophagy and survival and ultimately speed up osteoporosis

Limitation of this study is that other regulators existing in HSP60 immunoprecipitate may directly or indirectly modulate osteoblast autophagy to alter bone formation upon glucocorticoid stress. It also indicates that HSP60 plays an important role in dealing with the complex nature of intracellular organelle intercommunication during glucocorticoid-deregulated osteoblast behavior. Taken together, plausible investigations revealed that HSP60 is required to osteoblast survival and mineralized matrix anabolism compromising the development of glucocorticoid-mediated osteoporotic skeletons through sustaining RPTOR function to osteoblast autophagy as schemed in Fig. 8. This study highlights an emerging molecular mechanism by which chaperone HSP60 harmonizes organelle homeostasis within osteoblasts delaying the development of osteoporosis.

## Methods

### Glucocorticoid-stressed osteoblast cultures

Murine MC3T3-E1 osteoblasts were maintained in Dulbecco's Modified Eagle's Medium (DMEM) and 10% fetal bovine serum. 1 × 10^5^ cells/well were incubated in 24-well plates containing osteogenic medium (DMEM, 10% fetal bovine serum, 10 mM β-glycerophosphate, and 50 μg/ml ascorbic acid) (R&D System) with 1 μM dexamethasone or vehicle dimethyl sulfoxide for 18 days. Medium was changed every 3 days. Mineralized matrix deposits in cell cultures were detected using von Kossa staining, and area of mineralized nodules in three random fields in each well was measured using Zeiss Inverted microscopy and image analysis software, as previously described ^42^.

### RNA interference and cDNA transfection

cDNA coded human HSP60 or RPTOR were constructed into vectors pUSEamp( + ) (Upstate Biotechnolog, Lake Placid, NY). RNA interference targeting HSP60 (#11067) and RPTOR (#178055) were obtained from Applied Biosystems Biotechnology (ThermoFischer Scientific). Aliquots of 2 μg cDNA, RNAi or scramble controls were mixed with Lipofectamine^TM^ 3000 Transfection Reagent (Invitrogen^TM^; ThermoFischer Scientific) and transferred into cell cultures, according to the manufacturer’s instructions.

### Immunofluorescence analysis of HSP60, RPTOR, and autophagic puncta

After removing culture medium and rinsing with phosphate-buffered saline (PBS), cell cultures were fixed with 4% formaldehyde in PBS and blocked with normal goat serum (Cell Signaling Technology, Danvers, MA). HSP60 and RTPOR immunoreactivity in cell cultures were probed using HSP60 (#ab3080, Abcam Cambridge, MA) and RPTOR antibodies (#2280, Cell Signaling Technology, Danvers, MA). Goat anti-mouse IgG conjugated Alexa Fluor^®^ 488 and donkey anti-mouse IgG conjugated Alexa Fluor^®^ 675 (Abcam, Cambridge, MA) were used as secondary antibodies. After counterstaining with 4′,6-diamidino-2-phenylindole (DAPI) and covering with Prolong Gold Antifade Reagent (Cell Signaling Technology, Danvers, MA), immunofluorescence was evaluated using an Olympus Laser Confocal Microscope system. In some experiments, autophagic puncta in cell cultures were detected using fluorescent monodansylcadaverin probe of Autophagy Detection Kits (Abcam, Cambridge, MA), according to the manufacturer’s instructions. Number of puncta in 30 cells in three random fields of each well were counted.

### RT-quantitative PCR analysis of mRNA expression

Cell cultures were mixed with Trizol reagent to extract total RNA. Reverse transcription of 1 μg total RNA was performed using ReadScript^®^ Two-Step cDNA Synthesis Kits (Sigma-Aldrich). For PCR analysis, 2 × TaqMan^®^ Universal PCR Master Mix along with primers for probing HSP60 (forward, 5′-CGTTGCCAATAACACAAACG-3′; reverse, 5′-CGTTGCCAATAACACAAACG-3′), RPTOR (forward, 5′-GCAGAGCTGGAGAATGAAGG-3′; reverse, 5′-GTCGAGGCTCTGCTTGTA CC-3′), Atg4 (forward, 5′-CCAGCTTCAGCAAGATCTCC-3′; reverse, 5′-ATA CATCCCCAGCCACAGTC-3′), Atg12 (forward, 5′-CCAGCCCAATAGGACTC TTTAAC-3′; reverse, 5′-CACAGCACCGAAATGTCTC-3′), Runx2 (forward, 5′-CCAGCAGCACTCCATATCTC-3′; reverse, 5′-CAGCGTCAACACCATCATTC -3′), collagen 1α1 (forward, 5′-CACCCTCAAGAGCCTGAGTC-3′; reverse, 5′- CA GACGGCTGAGTAGGGAAC-3′), osteocalcin (forward, 5′-CAAGCAGGGAGGCA ATAAGG-3′; reverse, 5′-CGTCACAAGCAGGGTTAAGC-3′), and actin (forward, 5′-GACGGCCAGGTCATCACTAT-3′; reverse: 5′-CTTCTGCATCCTGTCAGCA A-3′) were mixed with the reverse transcription products. Gradient thermal programs and threshold values (Ct) computation were performed using a ABI 7900 Detection System (Applied Biosystems). Expressions of mRNA were calculated using the equation 2^-ΔΔCt^, where ΔCt resembled the difference in Ct values between the gene of interest and actin, and ΔΔCt stood for the difference in ΔCt between the glucocorticoid and vehicle group.

### Assessment of immunoblotting, protein aggregation, and immunoprecipitation

Lysates of 5 × 10^6^ cells were prepared using Mammalian Cell Extraction Kits (Abcam, Cambridge, MA). HSP60, RPTOR, Atg4 (#ab108322, Abcam, Cambridge, MA), Atg12 (sc-68884, Santa Cruz Biotechnology), LC3 (#PA1-16930, ThermoFischer Scientific), phosphorylated ERK (#9106), phosphorylated Ser473-Akt (#9721), phosphorylated RPTOR (#2083), Bax (#2774), cytochrome c (#12963), caspase3 (#9662), ubiquitin (#3936), and actin (#4967, Cell Signaling Technology, Danvers, MA) antibodies were used for probing the designated proteins in lysates using immunoblotting protocols. In some experiments, protein aggregates in 100 μg cell lysates were isolated after ultrahigh centrifugation at 10,000 g at 4 ℃ for 30 min, as previously described^20^. For positive controls, 100 μg cell lysates were mixed with equal volume of ice-cold acetone. Pellets were separated by electrophoresis and stained using Coomassie blue, and RPTOR levels in the pellet was probed using immunoblotting protocols. In addition, HSP60 and RPTOR immunoprecipitates were isolated for immunoblotting probed using ubiquitin antibody.

### Liquid chromatography and tandem mass spectrometry

Aliquots of HSP60 immunoprecipitates were electrophoresed, and protein bands of interest in the gel were cut for trypsin hydrolysis, as previously described^19^. Peptides in the hydrolyte were eluted through an Acclaim PepMap 100 column using a high-performance liquid chromatography system (Dionex Ultimate 3000, Dionex Corp., Sunnynale, CA). Peptide sequences were characterized using electrospray ionization mass spectrometry (Bruker Daltonik GmBH, Leipzig, Germany). Analytic results detected by tandem mass spectrometry was submitted to SWISS-PORT bioinformation resource portal (http://www.expaysy.com) to match the peptide homology.

### Proximity ligation assessment of HSP60 and RPTOR interaction

Interaction between HSP60 and RPTOR in cell cultures was probed using Duolink^®^ PLA Fluorescence Kits (Sigma-Aldrich). In brief, formaldehyde-fixed cell cultures were blocked using Duolink^®^ Blocking Solution and mixed with HSP60 and RPTOR antibodies, and secondary antibody Duolink® PLA probe. Specimens were incubated in a Duolink^®^ ligation buffer containing 1 U/μl ligase at 37 ℃ for 30 min and followed by reacting with an amplification solution containing 2 U/ml polymerase and counterstaining with DAPI. Fluorescent spots in cell cultures were detected using a Zeiss fluorescence microscope, and number of fluorescent spots were counted using image analysis system (Carl Zeiss, Gottingen, Germany).

### Lentivirus-shuttled RPTOR gene transfer for glucocorticoid-treated mice

Animal use protocols were reviewed and approved by IACUC, Kaohsiung Chang Gung Memorial Hospital (IACUC Affidavit No. 2011062101). HEK293 cells were transferred with 1 μg pMIF-cGFP-zeo plasmid coding RPTOR along with 1 μg pPACKF1 plasmid (System Biosciences, Palo Alto, CA). Culture supernatants were harvested, ultrahigh centrifuged, and titrated using LentiX qRT-PCR Titration Kits (Clontech Laboratories Inc, San Francisco, CA) to prepare 2 × 10^9^ infectious units/ml lentivirus particle suspension. Twelve-week-old male FVB mice were anesthetized through inhale isoflurane. Twenty-four mice were evenly divided into two groups and anesthetized to receive 50 μl RPTOR or mock lentivirus particle suspension through tail vein injection. One week after administration, mice were intra-peritoneally injected with 10 mg/kg/day methylprednisolone for 4 weeks. For control group, 12 mice were injected with normal saline only. At the end of experiment, mice were killed to dissect femurs and tibiae for study. Six mice were used for μCT and histological assay, and 6 animals for harvesting bone extract for immunoblotting.

### HSP60 transgenic mice

HSP60 transgenic mice (FVB/NNarl-TgPGK-HSP60; HSP60Tg) were bred, as previously described^18^. Siblings that did not express the construct were designated as wild-type mice. 36 male HSP60Tg and 36 wild-type mice (12-week old) were evenly divided into two groups to receive 10 mg/kg/day methylprednisolone or vehicle for 4 weeks. After euthanasia, bone tissue was dissected for study. Six mice were used for μCT imaging and histomorphology, six mice for harvesting total RNA in bone tissue, and six animals for isolating bone marrow cells.

### μCT assessment of bone mass and microstructure

Tibiae were X-ray scanned using a Skyscan 1176 μCT system (Bruker, Kontich, Belgium), as previously described^19^. BMD (g/cm^3^), BV/TV (%), Tb.Th (mm), Tb.N, and Ct.Th (mm) of 200 slices of images with 9 μm voxel size between growth plate region and midshaft region of proximal tibiae were calculated using SKYSCAN^®^ CT-Analysis software.

### Immunohistochemistry and histomorphometry

Sections of methylacrylate-embedded tibiae blocks were stained using von Kossa reagent. Fluorescent calcein intensity was evaluated using a fluorescence microscope. Three sections of each animal were selected for measuring BV/TV (%) and mineral apposition rate (MAR; μm/day). Immunohistochemical assessment was performed using LC3 antibody and BioGenex Immunohistochemistry Detection Kits (BioGenex, Fremont, CA). TUNEL staining in sections was probed using In Situ TUNEL Detection Kits (Roche Diagnostics GmbH). Osteoblasts showing positive immunostaining or TUNEL staining in each field were counted. Three fields in each section, and three sections of each mice were selected for measurement.

### Ex vivo osteogenic differentiation of bone marrow mesenchymal cells

Primary bone marrow mesenchymal cells were isolated, as previously described^19^. In total, 1 × 10^5^ cells/well bone marrow mesenchymal cells were incubated in 24-well plates containing osteogenic medium for 18 days. Mineralized matrix formation was stained using von Kossa staining reagents, and area of mineralized matrix in three random fields in each well was measured. In some experiments, cell cultures were harvested for reverse transcription polymerase chain reaction assessment of osteogenic gene expression.

### Statistical analysis

Experiments of cell culture models were repeated five times. Parametric analysis of variance and Bonferroni post hoc test were utilized to examine whether experimental results among vehicle, mock-treated, and RPTOR-treated groups, as well as the investigations among vehicle- and glucocorticoid-treated HSP60Tg mice and wild-type mice were significant different as the *P* values were set at <0.05.
