# Correction: Chaperonin 60 sustains osteoblast autophagy and counteracts glucocorticoid aggravation of osteoporosis by chaperoning RPTOR

**DOI:** 10.1038/s41419-019-1991-5

**Published:** 2019-10-03

**Authors:** Wei-Shiung Lian, Jih-Yang Ko, Yu-Shan Chen, Huei-Ching Ke, Shin-Long Wu, Chung-Wen Kuo, Feng-Sheng Wang

**Affiliations:** 1 Core Laboratory for Phenomics and Diagnostic, Kaohsiung Chang Gung Memorial Hospital, Kaohsiung, Taiwan; 2 Department of Medical Research, Kaohsiung Chang Gung Memorial Hospital, Kaohsiung, Taiwan; 3 Department of Orthopedic Surgery, Kaohsiung Chang Gung Memorial Hospital, Kaohsiung, Taiwan; 4 Graduate Institute of Clinical Medical Science, Chang Gung University College of Medicine, Kaohsiung, Taiwan

**Correction to:** **Cell Death & Disease**

10.1038/s41419-018-0970-6, published online 17 September 2018

Following publication of this article, the authors realized that there were 1) errors made in the author affiliations and that 2) a typo in a grant number needed to be corrected. The corrected author affiliations and grant numbers are listed below. We apologize for the inconvenience.

1) Wei-Shiung Lian^1,2^, Jih-Yang Ko^3^, Yu-Shan Chen^1,2^, Huei-Ching Ke^1,2^, Shin-Long Wu^1,2^, Chung-Wen Kuo^1,2^, Feng-Sheng Wang^1,2,4^

1 Core Laboratory for Phenomics and Diagnostic, Kaohsiung Chang Gung Memorial Hospital, Kaohsiung, Taiwan

2 Department of Medical Research, Kaohsiung Chang Gung Memorial Hospital, Kaohsiung, Taiwan

3 Department of Orthopedic Surgery, Kaohsiung Chang Gung Memorial Hospital, Kaohsiung, Taiwan

4 Graduate Institute of Clinical Medical Science, Chang Gung University College of Medicine, Kaohsiung, Taiwan

2) In the Acknowledgements section, grant numberCMRPG8H00112 is incorrect. The correct grant number is CMRPG8H0111.

This has been corrected in both the PDF and HTML versions of the Article.

